# Genomic and phenotypic characterization of *Pseudomonas* sp. GOM7, a novel marine bacterial species with antimicrobial activity against multidrug-resistant *Staphylococcus aureus*

**DOI:** 10.1371/journal.pone.0288504

**Published:** 2023-07-13

**Authors:** Luis E. Romero-González, Jorge Rojas-Vargas, Luis F. Muriel-Millán, Jaime Bustos-Martínez, Víctor H. Bustamante, Liliana Pardo-López

**Affiliations:** 1 Departamento de Microbiología Molecular, Instituto de Biotecnología, Universidad Nacional Autónoma de México, Cuernavaca, Morelos, México; 2 Departamento de Atención a la Salud, Universidad Autónoma Metropolitana Unidad Xochimilco, CDMX, México; University of Hertfordshire hosted by Global Academic Foundation, EGYPT

## Abstract

Antimicrobial resistance (AMR) represents a serious threat to global health. The development of new drugs to combat infections caused by bacteria resistant to multiple or even all available antibiotics is urgent. Most antibiotics used up to date have been identified from soil microorganisms. The marine environment represents an alternative source with great potential for the identification of microorganisms that produce bioactive molecules, including antibiotics. In this study, we analyzed the antibacterial activity of a collection of 82 bacterial strains isolated from marine water and sediment samples collected from the Southwestern Gulf of Mexico. Eight of the marine isolates inhibited the growth of different pathogenic bacteria, seven of which were identified as presumptive *Pseudomonas aeruginosa*. Interestingly, genome sequencing and phylogenetic analysis revealed that the remaining marine isolate showing antibacterial activity is a novel *Pseudomonas* species that we denominated *Pseudomonas* sp. GOM7, which was not pathogenic in the *Galleria mellonella* infection model in the conditions tested. Notably, *Pseudomonas* sp. GOM7 inhibited the growth of multidrug and methicillin-resistant strains of the priority pathogen *Staphylococcus aureus*. Our results show that the anti-*S*. *aureus* compound(s) produced by *Pseudomonas* sp. GOM7 can be extracted from the culture supernatant of this bacterium with the organic solvent ethyl acetate. Annotation of the *Pseudomonas* sp. GOM7 genome revealed the presence of several biosynthetic gene clusters predicted to code for possible antimicrobial compounds. Our results further highlight the potential of bacteria from the Gulf of Mexico as a source of novel antimicrobials.

## Introduction

Currently, antimicrobial resistance (AMR) represents a serious threat to global health. The presence of bacteria resistant to several or even all commercial antibiotics makes treating infectious diseases very challenging and increases mortality and hospitalization expenses [1]. The crisis by AMR has been attributed to numerous factors, including the widespread use of antibiotics in humans, farm animals, and agriculture, as well as incorrect antibiotic prescription practices and a decline in the discovery of novel drugs [2]. Additionally, an increase in the proliferation of antibiotic-resistant bacteria is anticipated as a result of the high consumption of antibiotics, disinfectants, and biocide agents during the COVID-19 pandemic [3, 4]. In 2017, the World Health Organization (WHO) published a list of 12 bacteria denominated as priority pathogens, which present resistance to the antibiotics commonly used to combat their infections; thus, it is urgent to develop new drugs against these pathogenic bacteria. One of these priority pathogens is the methicillin-resistant *Staphylococcus aureus* (MRSA), which also presents resistance to multiple other antibiotics [5]. MRSA is a prevalent cause of hospital-acquired infections, including pneumonia, bacteremia, and skin infections, and it is associated with high morbidity and mortality [6]. For instance, Centers for Disease Control and Prevention (CDC) assessments estimated 119 000 infections and 20 000 deaths from invasive MRSA infections in 2017 in the US. The burden of MRSA has increased over the years, resulting in a substantial healthcare cost estimated at US$ 450 million in the last decade. Furthermore, *S*. *aureus* is the most common cause of coinfection in patients with COVID-19, and MRSA ratios have increased during the pandemic [7, 8].

Most antibiotics are produced by microorganisms; approximately 80 antibacterial drugs that were licensed between 1981 and 2014 are either natural compounds or directly derived from primary or secondary metabolites produced by microorganisms [9, 10]. Soil microbes have been the main source of antibiotics; however, further attempts to identify new antibiotics from this source have largely rediscovered known compounds [11]. Searching for new antibacterial compounds is now being directed to microorganisms present in poorly explored sources, such as animals, plants, caves, and bodies of water [12]. In particular, the oceans, covering over 70% of the Earth’s surface and containing a high diversity of microorganisms, represent a promising source of bioactive compounds. Marine bacteria are exposed to variations in environmental conditions, such as temperature, pressure, light, and salinity, which favor the production of unique compounds [13]. Molecules produced by marine bacteria include alkaloids, steroids, terpenoids, peptides, and polyketides [14]. The Gulf of Mexico (GoM) is the ninth largest body of water in the world, bordered by the USA on the northern and eastern sides, by Mexico on the western and southern sides, and by Cuba on the southeast side; it covers a surface area of more than 1.5 million km^2^. Recent studies have shown the diversity of bacterial communities from the GoM and their role in different biogeochemical cycles [15]. A bacterial genus found abundantly in the GoM is *Pseudomonas* [16]. *Pseudomonas* species are present in different environments such as water, soil, animals, and plants [17]. Furthermore, *Pseudomonas* species show a high genomic plasticity that results in extraordinary metabolic versatility, producing a plethora of bioactive secondary metabolites, including phenazine derivatives like pyocyanin [18, 19].

In this study, we explored a collection of marine bacterial isolates as a source of possible new antibiotics. We identified a novel species of *Pseudomonas*, *Pseudomonas* sp. GOM7, which shows growth inhibitory activity against multidrug resistant (MDR) *S*. *aureus* strains, including the priority pathogen MRSA.

## Materials and methods

### Bacterial strains and growth conditions

The eighty-two marine bacteria tested were previously isolated from sediments and seawater samples from the GoM [20]. *Pseudomonas* sp. GOM7 was obtained from a seawater sample collected at a depth of 55 m (25° 38.199′ N; 95°1.283′ W) [15, 20]. The nonmarine bacterial strains used in this study are described in S1 Table. *S*. *aureus* isolates were obtained from healthy young adults (18–21 years old) in the period from 2019 to 2021. Samples were taken from nasal or pharyngeal exudates and phenotypically identified using an API®Staph system (BioMérieux, Marcy l’Étoile, France). Reference strains tested in this study were obtained from the American Type Culture Collection (ATCC; Manassas, VA). All bacterial strains were cultivated in lysogeny broth (LB) or Mueller-Hinton (M-H) broth at 37°C or 30°C.

### Ethics statement

Sampling protocol for *S*. *aureus* isolation was approved by the Ethics Committee of the Biological Sciences and Health Division of the UAM-Xochimilco (Document: DCBS.CD.056.18). All participants provided their written informed consent to participate as volunteers. No incentives were offered.

### Antibiotic susceptibility

The antibiotic susceptibility of *S*. *aureus* isolates was determined by the Kirby-Bauer disk diffusion method according to CLSI recommendations [21]. The antibiotics tested were ciprofloxacin (CIP), fosfomycin (FOS), trimethoprim/sulfamethoxazole (TRS), penicillin (P), vancomycin (VAN), tetracycline (TET), erythromycin (ERY), clindamycin (CLI), gentamicin (GEN), and cefalotin (CEF). Methicillin resistance was evaluated by obtaining the minimal inhibitory concentration (MIC) for oxacillin (OXA) using the previously described microdilution assay [22]. Antibiotics were purchased from Sigma-Aldrich. The antibiotic susceptibility profiles found for the *S*. *aureus* isolates are indicated in S2 Table.

### Organic extraction of the culture supernatant

Organic crude extracts from the supernatant of bacterial cultures were obtained by extraction with solvents of different polarities (*n*-hexane, chloroform, and ethyl acetate). Culture supernatants were obtained by growing *Pseudomonas* sp. GOM7 or *E*. *coli* DH10β in 200 ml of diluted (1:4) LB for 48 h at 30°C with aeration. Cells were removed by centrifugation at 8,586 *g* for 30 min at 4°C. Then, 100 ml of the supernatant was extracted with the respective solvent in a 5:1 ratio. The mixture (supernatant and solvent) was shaken vigorously for 10 min and allowed to stand for 5–10 min. The organic layer was collected, and the extraction was repeated two more times with the addition of fresh solvent. The organic phase was dried at room temperature in a fume hood and the powder obtained was stored until use. The organic extracts were solubilized in a sterile 10% dimethyl sulfoxide (DMSO) solution to a final concentration of 0.6 g ml^-1^.

### Antibacterial activity assays

The growth inhibitory effect on bacteria was assessed by the colony method. Indicator strains (assessed for growth inhibition) were grown in LB at 37˚C up to an optical density at 600 nm of 0.6 (≈ 1 x 10^8^ CFU ml^-1^). LB agar plates were inoculated with 5 ml of each indicator strain culture. After 5 min, the cultures were removed and the plates were dried under sterile conditions. Producer bacteria (assessed for production of antibacterial activity) were grown overnight in LB at 37°C. Then, 3 ml of these cultures were concentrated by centrifugation and resuspended in 50 μl of sterile LB. A 10 μl drop of the producer bacteria suspensions was placed at equidistant points in the previously inoculated LB agar plates. The plates were incubated for 24 h at 37°C. The *E*. *coli* DH10β strain was tested as a negative control of producer bacteria.

Antibacterial activity of bacterial culture supernatants was assessed as follows. Culture supernatants from *Pseudomonas* sp. GOM7 or *E*. *coli* DH10β were obtained as described above; then, the supernatant was lyophilized in a Labconco™ FreeZone™ 2.5 Benchtop Freeze Dry System. The obtained powder was rehydrated with 5 ml of sterile Milli-Q water; it was thus concentrated 40 times. The antibacterial activity of the supernatants was determined by an agar-well assay [23] on M-H agar plates, which were inoculated with indicator bacteria as described before. The top part of a 1 ml sterile micropipette tip was used to create wells in the inoculated agar plates. Then, 100 μl of the resuspended lyophilized supernatant was loaded in each well, and the plates were incubated for 24 h at 30°C. The presence of an inhibition halo was considered positive for antibacterial activity in these assays.

Bacterial viability in organic extracts was assessed by following previously described methods to evaluate antibacterial activity of conditioned media, with some modifications [24, 25]. *S*. *aureus* ATCC 43300 was grown in LB up to an optical density at 600 nm of 0.6 (≈ 1 x 10^8^ CFU ml^-1^); then, 10 μl of this culture were inoculated into 1 ml of the solubilized organic extract and the samples were incubated at 37°C for 5 h. Colony forming units (CFUs) were quantified at 0, 30, 60, 120, 180, and 300 min post-inoculation by plating on LB agar plates. Organic extracts from *E*. *coli* DH10β strain and growth in LB medium were used as negative control in these assays.

### Biofilm formation

Biofilm formation was evaluated by the crystal violet assay in 96-well polystyrene microtiter plates (Costar®, Corning Incorporated) as previously described [26]. Bacterial strains were grown in LB for 24 h at 37°C. Absorbance lectures at 570 nm were determined in a microtiter plate reader (BioTek™ Epoch2™, San Diego, CA, USA).

### Pyocyanin production

Observation of pyocyanin production was performed by growing the bacterial strains on cetrimide agar; bacteria that form green or blue colonies are considered to produce pyocyanin. Quantification of pyocyanin from the supernatant of bacterial cultures grown in LB was determined as previously reported [27]. Concentration was calculated by multiplying absorbance with the specific molar extinction coefficient of the pyocyanin.

### Protease assay

Protease activity was detected as clearing zones on skim-milk agar plates [28].

### Pathogenicity assays

Pathogenicity was analyzed using the *Galleria mellonella* (wax moth) infection model as described previously [29]. Groups of ten *G*. *mellonella* larvae were inoculated through the lower left proleg with 10 μl of a bacterial suspension in 1X PBS containing 0.5x10^3^-1x10^6^ CFU ml^-1^, using an insulin syringe. Infected larvae were incubated at 30°C without food and inspected at 4, 7, 20, 25, 31, 44, 68, 98, and 116 h post-injection to record mortality.

### Purification and sequencing of DNA

Genomic DNA was purified using the Quick-gDNA miniprep kit from Zymo Research (Irvine, CA, United States) according to the manufacturer’s instructions from overnight bacterial cultures in LB at 37°C. For sequencing of the 16S rRNA gene, PCRs were performed using the purified genomic DNA, the universal primers fD1 and rP2 [30], and the GoTaq Flexi DNA polymerase (Promega). Amplification products were purified with the DNA Clean & Concentrator kit from Zymo Research (Irvine, CA, United States) and subsequently sequenced using the Sanger method at the DNA Synthesis and Sequencing Unit at the Institute of Biotechnology (IBt) / UNAM. Sequences were cleaned using Chromas v.2.6.6 (http://technelysium.com.au/wp/chromas/), assembled by CAP in BioEdit v7.1 [31], and annotated based on a BLASTN search of the NCBI database 16S ribosomal RNA sequences for Bacteria and Archaea [32]. For sequencing the *Pseudomonas* GOM7 genome, a combination of the platforms Illumina MiSeq (Illumina Inc., San Diego, CA, USA) and Oxford Nanopore Technologies MinION were used at the Massive Sequencing Unit of the IBt / UNAM. Illumina sequencing was performed using the Nextera library kit (Illumina, Inc.), and DNA was cut to generate fragments of an average size of 500 bp. A 600-cycle sequencing kit was used to obtain 556,352 paired-end reads with a length of 300 bp. The read quality was examined using FastQC v0.11.9 [33] and raw sequences were filtered with a quality ≥Q20 using Trimmomatic v0.39 [34]. For Nanopore sequencing, libraries were prepared with the SQK-LSK109 kit and multiplexed using the EXP-NBD104 barcoding kit. The libraries were loaded into the R9.4.1 flow cell. Reads were base called and demultiplexed using Guppy v4.4.1 [35], adapters were trimmed, and barcode separation was performed by Porechop v0.2.4 (https://github.com/rrwick/Porechop). The read quality was examined with NanoPlot v1.30.1 [36].

### Genome assembly and annotation

Genome *de novo* assembly was performed with Unicycler assembler v0.4.8 [37] using the default parameters. The quality analysis of the assembly was performed with QUAST v4.0 [38] and the completeness and contamination were evaluated with CheckM v1.1.3 [39]. The complete *Pseudomonas* sp. GOM7 genome was uploaded to the BV-BRC web server (https://www.bv-brc.org/) for gene prediction and functional annotation with the RASTtk pipeline [40]. The genome was also surveyed for secondary metabolite biosynthetic gene clusters using the antiSMASH online tool v6.1.1 [41], and comparison between genes was performed with the Clinker program [42].

### Taxonomic identification and phylogeny

To identify the closest genomes, the *Pseudomonas* sp. GOM7 genome was analyzed by GTDB-Tk v2.0.0 [43] using the GTDB R207 reference database with 317,542 genomes (April 9, 2022) and the de_novo_wf command. The closest GTDBtk genomes were retrieved from the NCBI database (consulted on March 13, 2023), and different phylogenomic criteria for delimiting species were calculated: the genome distance estimation (Mash-D < 0.05) by the Mash program [44], the average nucleotide identity based on MUMmer (ANIm > 95%) by JSpeciesWS [45], the average amino acid identity (AAI > 96%) values with the CompareM tool (https://github.com/dparks1134/CompareM), the digital DNA-DNA hybridization (dDDH > 70%) formula d4 and the G+C content percentage difference (< 1%), both by TYGS [46]. The outgroup genome was that of the *Azotobacter vinelandii* strain DSM 279 assembly (GCA_900119555.1), downloaded from the NCBI website (consulted on March 13, 2023).

### Comparative genome and genome synteny analyses

For comparative genomic analysis, the *Pseudomonas* sp. GOM7 genome and its closest *Pseudomonas* genomes derived from the phylogenomic criteria analysis were tested. The number of gene families shared by the genomes was obtained using the CMG BioTools software v2.2 [47]. For genome synteny analyses, Sibelia software v3.0.7 [48] was used with a minimum block size of 20,000 bp, and the alignment of syntenic blocks was visualized in Circos [49].

### Statistical analysis

Data are presented as the mean ± standard deviation. Statistical analyses were performed using GraphPad Prism version 8.0.1 software for Windows (GraphPad Software, San Diego, CA, USA). One-way ANOVA, combined with Dunnett’s multiple comparison test, or the log-rank test, were used as indicated. *P* values of <0.05 were considered statistically significant.

## Results

### Identification of *Pseudomonas* marine isolates with antibacterial activity

In a previous study we isolated a collection of bacteria from the GoM, which was tested for hydrocarbon degradation capacity [20]. Here, we analyzed this collection of marine bacteria as a source of possible antibiotics. A total of 82 bacterial isolates from the GoM were tested for growth inhibitory activity against the reference strains *Acinetobacter baumannii* ATCC 17978, *Salmonella enterica* serotype Typhimurium (*S*. Typhimurium) SL1344, *S*. *aureus* ATCC 29213, and *Escherichia coli* ATCC 25922 by the colony method. 74 isolates, including different genera such as *Staphylococcus*, *Enterobacter*, *Pseudomonas*, *Bacillus*, and *Chryseobacterium* did not show antibacterial activity. In contrast, seven isolates generated inhibition halos on all tested bacteria; six of these isolates were identified as *P*. *aeruginosa* by sequencing of the 16S rRNA gene (S3 Table), and the other one was previously identified as *P*. *aeruginosa* GOM1 by genome sequencing [20]. Consistently, these seven isolates were shown to produce the phenazine pigment pyocyanin (S4 Table), a trait of *P*. *aeruginosa* [50]. Because *P*. *aeruginosa* strains are recognized to present antibacterial activity by the production of pyocyanin and bacteriocins [51, 52], the referred isolates were not further analyzed. One other marine isolate, GOM7, generated inhibition halos mostly on *S*. *aureus* strains (Table 1). The best match to the 16S rRNA gene of the GOM7 isolate was the *P*. *sihuiensis* strain WM-2 (S3 Table). However, the genomic analysis described below indicated that the GOM7 isolate is actually a new *Pseudomonas* species, which we denominated *Pseudomonas* sp. GOM7. As expected, the production of pyocyanin was not detected in *Pseudomonas* sp. GOM7 (S1 Fig). These results revealed to *Pseudomonas* sp. GOM7 as an interesting subject for further analysis.

**Table 1:** Antibacterial activity spectrum of *Pseudomonas* sp. GOM7.

Strain tested	*Pseudomonas* sp. GOM7^a^		
	Colony	Supernatant	Ethyl acetate extract
**Gram-positive**			
***Staphylococcus aureus* ATCC 29213**	+	+	+
***S*. *aureus* ATCC 43300 (MRSA)**	+	+	+
***S*. *aureus* 8N2 (MRSA)**	+	+	+
***S*. *aureus* 4N34 (MRSA)**	+	+	+
***S*. *aureus* 14F4A (MRSA)**	+	+	+
***S*. *aureus* 25F4 (MRSA)**	+	+	+
***S*. *aureus* 15N4 (MRSA)**	+	+	+
***S*. *aureus* 1N3 (MRSA)**	+	+	n.d.
***S*. *aureus* 13F3 (MRSA)**	+	+	n.d.
***S*. *aureus* 21F3 (MRSA)**	+	+	+
***S*. *aureus* 6N3 (MDR)**	+	+	+
***S*. *aureus* 18F1 (MDR)**	+	+	+
***S*. *aureus* 24N2 (MDR)**	+	+	+
***S*. *aureus* 25F2 (MDR)**	+	+	n.d.
***S*. *aureus* 17F3 (MDR)**	+	+	+
***S*. *aureus* 17N3 (MDR)**	+	+	+
***B*. *subtilis* 168**	+	-	n.d.
***Enterococcus faecium* ATCC 19434**	-	-	n.d.
**Gram-negative**			
***E*. *coli* ATCC 25922**	+	-	n.d.
***S*. Typhimurium SL1344**	+	-	n.d.
***Klebsiella quasipneumoniae* ATCC 700603**	-	-	n.d.
***A*. *baumannii* ATCC 17978**	-	-	n.d.

^a^Antibacterial activity was observed by the formation (+) or not (-) of an inhibition halo on the strain tested; n.d. not determined. The *E*. *coli* DH10β strain, used as a negative control, did not exhibit antibacterial activity neither by colony nor by the supernatant or ethyl acetate extract. LB and M-H agar plates were used to analyze the effect of the colonies or culture supernatants and ethyl acetate extract respectively, which were incubated for 24 h at 37°C for colony assays or 30°C for supernatant and ethyl acetate extract analysis. Assays were performed in triplicate.

### *Pseudomonas* sp. GOM7 shows inhibitory activity against *S*. *aureus* strains resistant to antibiotics

**Fig 1:**
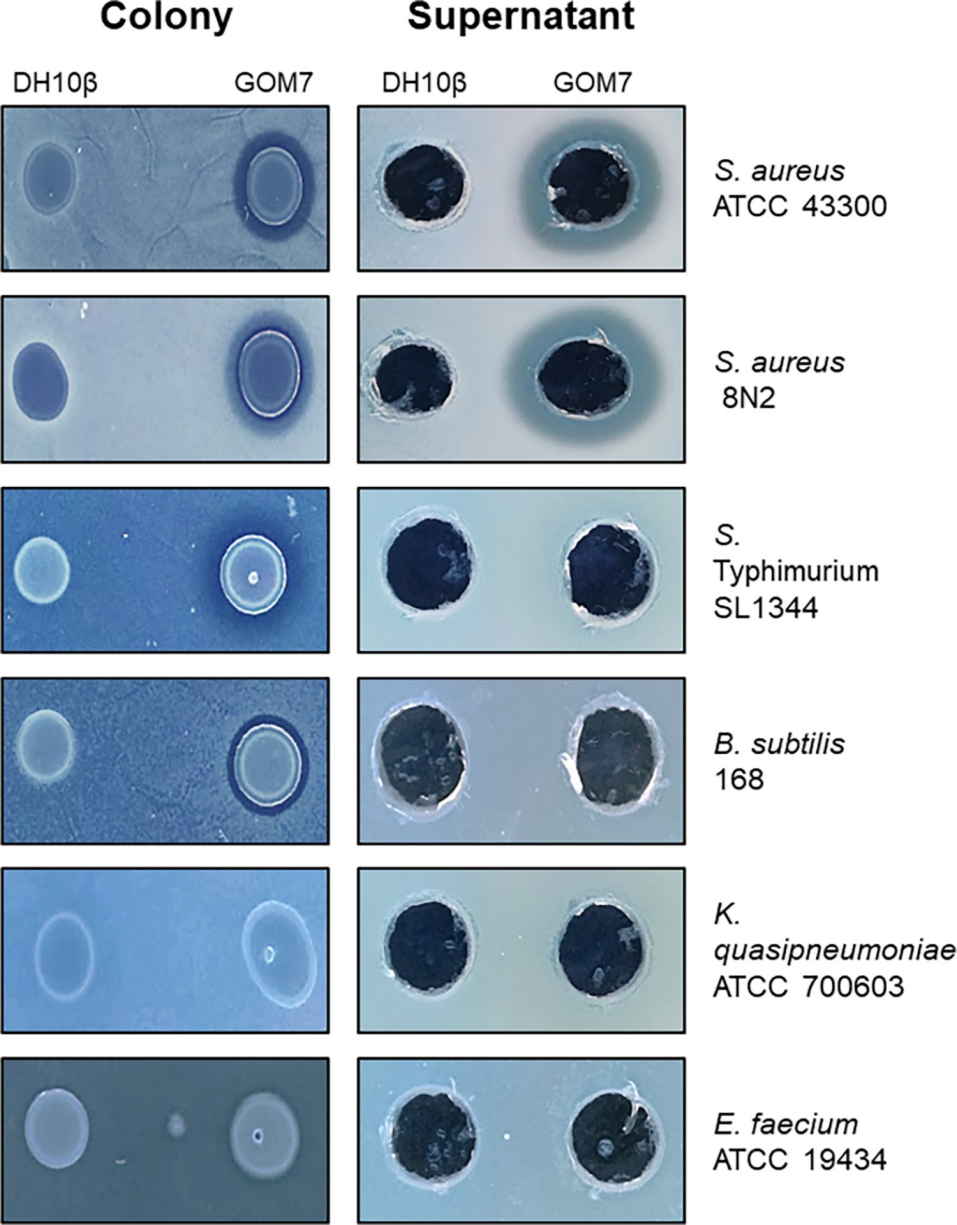
Inhibition halos formed by *Pseudomonas* sp. GOM7. Colonies or the culture supernatant of *Pseudomonas* sp. GOM7 were tested for formation of inhibition halos on different bacteria. The *E*. *coli* DH10β strain was used as a negative control. Images are representative of the results shown in Table 1.

Antibacterial activity of *Pseudomonas* sp. GOM7 was further analyzed against different bacteria including antibiotic-resistant strains of *S*. *aureus*. Of note, both the colonies and the culture supernatant (concentrated by lyophilization) of *Pseudomonas* sp. GOM7 generated inhibition halos on all the antibiotic-resistant strains of *S*. *aureus* tested (Table 1). In contrast, only the colony of *Pseudomonas* sp. GOM7 generated inhibition halos on *Bacillus subtilis* 168, *S*. Typhimurium SL1344, and *E*. *coli* ATCC 25922, and neither the bacterial colony nor the culture supernatant generated inhibition halos on *E*. *faecium* ATCC 19434, *K*. *quasipneumoniae* ATCC 700603, and *A*. *baumannii* ATCC 17978 (Table 1). As expected, neither the bacterial colony nor the culture supernatant of the *E*. *coli* DH10β strain, used as a negative control, formed inhibition halos on the bacteria tested (Table 1). Representative images for the formation or not of inhibition halos by the *Pseudomonas* sp. GOM7 and *E*. *coli* DH10β strains are shown in Fig 1. These results support that *Pseudomonas* sp. GOM7 produces one or more compounds with antibacterial activity.

**Fig 2:**
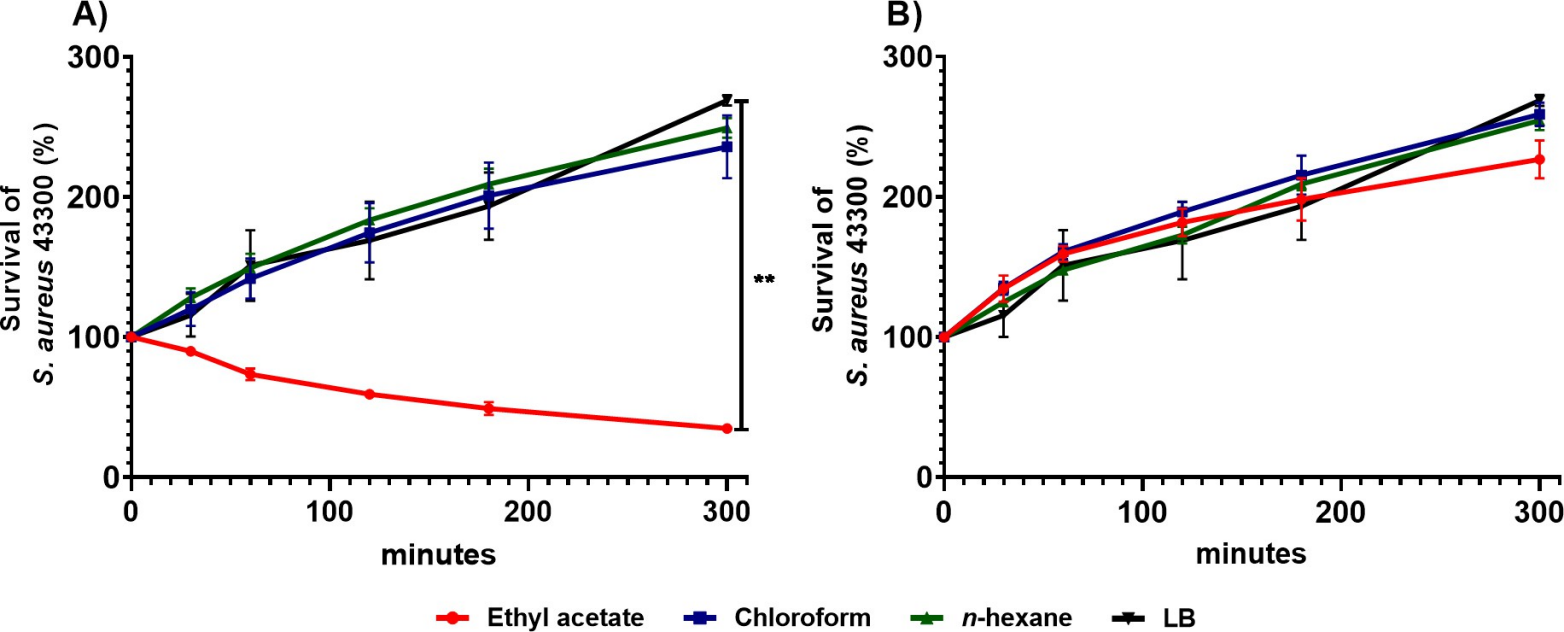
Ethyl acetate crude extract from *Pseudomonas* sp. GOM7 reduces the survival of MRSA. Approximately 1 x 10^6^ CFUs of *S*. *aureus* ATCC 43300 were inoculated into LB (black) (positive control of viability and growth), or into *n*-hexane (green), chloroform (blue), or ethyl acetate (red) extracts obtained from the culture supernatant of *Pseudomonas* sp. GOM7 (A) or *E*. *coli* DH10β (B). At the indicated times, CFUs were counted on LB agar plates and then bacterial survival percentage values were calculated and graphed. *P* value was calculated using One-way ANOVA combined with Dunnett’s multiple comparison test. * *P* <0.05, ** *P* <0.01, *** *P* <0.001. Data are expressed as the mean ± S.D. of three independent experiments.

To gain insight on the solubility of the compounds with antibacterial activity produced by *Pseudomonas* sp. GOM7, we tested crude extracts from the culture supernatant of this bacterium obtained with different organic solvents: As negative controls, crude extracts from the culture supernatant of *E*. *coli* DH10β obtained with the same solvents were also assessed in these assays. Only the ethyl acetate extract from *Pseudomonas* sp. GOM7 formed inhibition halos on *S*. *aureus*, including MRSA strains (Table 1). Additionally, we analyzed the survival of the *S*. *aureus* ATCC 43300 strain (MRSA) when exposed through time to ethyl acetate, *n*-hexane or chloroform extracts from *Pseudomonas* sp. GOM7 or *E*. *coli* DH10β. Notably, the ethyl acetate extract from *Pseudomonas* sp. GOM7 reduced ~60% the survival of *S*. *aureus* after 5 h of exposition; in contrast, bacteria replicated in the *n*-hexane and chloroform extracts (Fig 2A). As expected, no inhibitory effect was observed with the solvent extracts from *E*. *coli* DH10β (Fig 2B).

Collectively, these results indicate that *Pseudomonas* sp. GOM7 secretes one or more compounds that affect the viability of MDR *S*. *aureus* and importantly the priority pathogen MRSA.

### Sequencing, assembly, and annotation of the *Pseudomonas* sp. GOM7 genome

**Fig 3:**
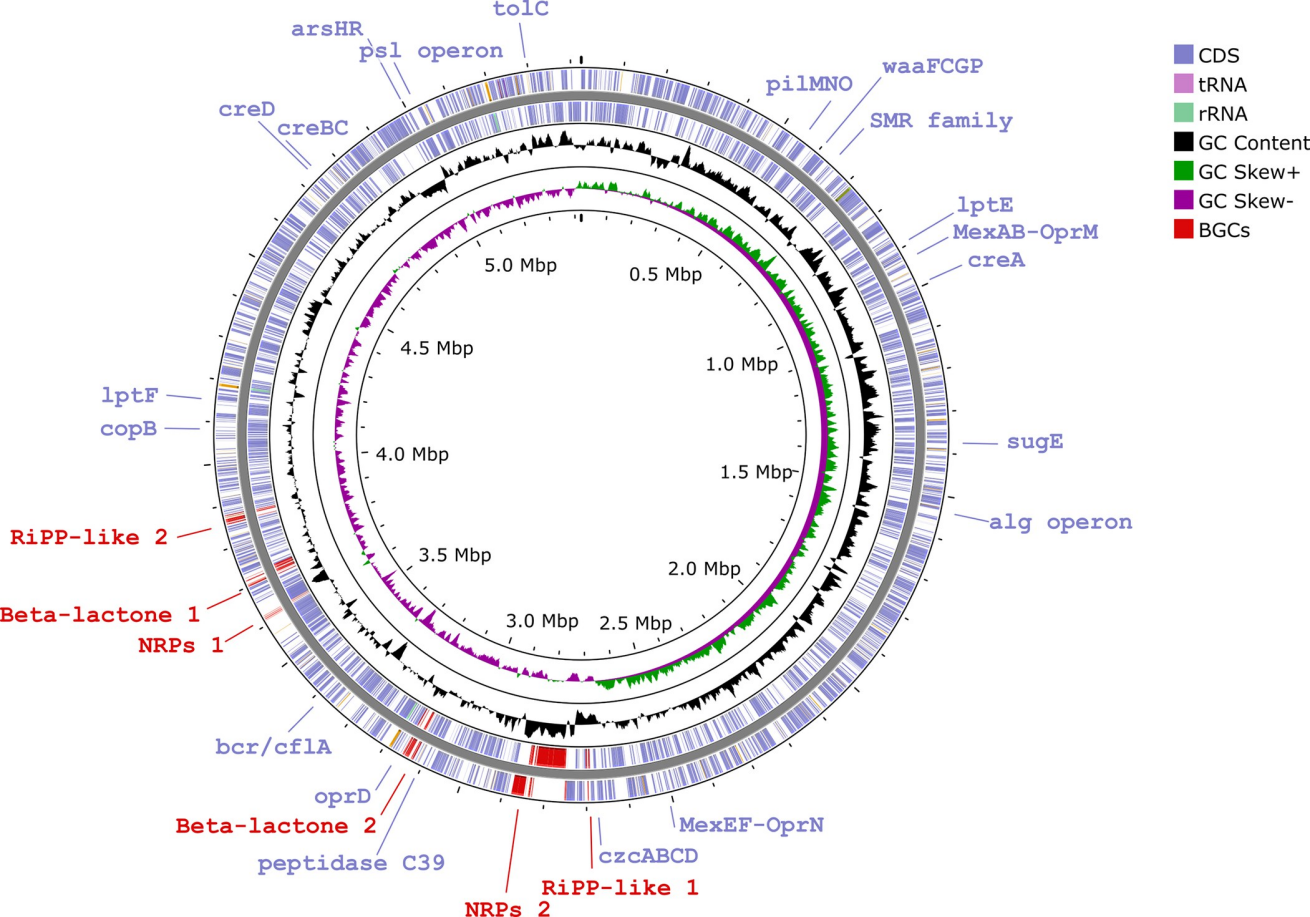
Circular representation of the *Pseudomonas* sp. GOM7 genome. The contents of the featured rings (starting with the outermost ring to the center) are as follows: (blue square) CDS forward and CDS reverse, (black square) GC content, (green square, purple square) GC skew, and genome size. Some genes annotated as encoding virulence factors, antimicrobial resistance, and potential resistance to other compounds are indicated in blue. Biosynthetic gene cluster (BGC) regions predicted by antiSMASH to encode potential antimicrobial compounds are shown in red (red square). The figure was produced using the CGView Server.

To determine the genetic characteristics of *Pseudomonas* sp. GOM7, we sequenced its whole genome using a hybrid approach with Illumina MiSeq and Nanopore MinION platforms. Illumina sequencing resulted in 556,352 paired-end reads with a median length of 300 bp and a median sequence quality (Phred score) of 36. The MinION device generated 39,185 reads with a mean length of 15,917 nucleotides (nt), a maximum read length of 133,102 nt, an N50 value of 23,048 nt, and a mean read quality of 13. For the assembly, completeness of 100% and contamination of 0.40% were obtained. The complete circular chromosome contains 5,424,934 bp in 1 contig and has a G+C content of 63.35% (Fig 3). A total of 4,774 coding sequences were predicted using RASTtk annotation. The genome harbors genes for 64 tRNA and 12 rRNA, as well as 63 CRISPR repeats. Four copies of the 16S rRNA gene were found, each with a length of 1,532 bp.

The whole-genome sequence data and raw sequences of the *Pseudomonas* sp. GOM7 are available at NCBI under accession number CP113519, BioProject accession number PRJNA905872, and Sequence Read Archive (SRA) accession numbers SRX18440904 (Illumina raw sequence data) and SRX18440905 (MinION raw sequence data).

### *Pseudomonas* sp. GOM7 is a new species of *Pseudomonas*

Whole genome sequence analyses including average nucleotide identity (ANI) and digital DNA-DNA hybridization (dDDH) have proven to be reliable approaches for phylogenomic studies and species definition [53, 54]. For the *Pseudomonas* genus, a multi-locus sequence analysis (MLSA) based on sequence comparison of the 16S rRNA, *gyrB*, *rpoB*, and *rpoD* genes has shown results comparable to those from whole genome sequence analyses to determine phylogenetic relationships [55, 56]. To better identify the species of *Pseudomonas* sp. GOM7, we first performed a GTDB-Tk analysis based on the concatenated alignment of 120 bacterial genes, including the 16S rRNA, *gyrB* and *rpoB* genes, from the *Pseudomonas* sp. GOM7 genome and all the genomes from the GTDB-Tk database. This analysis revealed 25 *Pseudomonas* species as the closest relatives to *Pseudomonas* sp. GOM7 (S2 Fig). Then, five phylogenomic criteria were calculated using the genome of *Pseudomonas* sp. GOM7 as the query, the genomes of the closest *Pseudomonas* species as the subject, and the genome of *Azotobacter vinelandii* DSM 279 as the outgroup (Table 2). In this analysis, a subject genome should meet all the calculated phylogenomic criteria (dDDH >70%, ANI% >96%, AAI >96%, Mash D <0.05, and G+C content difference <1%) to assign the respective species to the query genome [44, 46, 57]. However, none of the subject genomes analyzed met these criteria; only a few genomes yielded a G+C content difference lower than 1% (Table 2). Thus, *Pseudomonas* sp. GOM7 could not be classified as a known *Pseudomonas* species.

**Table 2:** Phylogenomic criteria to identify taxonomically the species of *Pseudomonas* sp. GOM7.

Genome	dDDH (d4, %)	ANIm (%)	AAI (%)	Mash D	G+C content difference (%)
***P*. *guguanensis* JCM 18416 (GCA_900104265.1)** ^**a**^	31.3	88.30	88.19	0.11	0.86
***P*. *composti* LY1 (GCA_001567565.1)**	31.2	88.25	88.59	0.12	0.36
***Pseudomonas* sp. P818 (GCA_000418555.1)**	30.9	88.17	88.24	0.12	0.07
***P*. *indoloxydans* JCM 14246 (GCA_003052605.1)** ^**a**^	30.8	88.00	87.94	0.11	1.10
***P*. *mendocina* EF27 (GCA_008041835.1)**	30.8	87.96	87.71	0.12	1.56
***P*. *oleovorans subsp*. *oleovorans* (GCA_002197815.1)** ^**a**^	30.7	87.90	88.26	0.11	1.29
***P*. *alcaliphila* JAB1 (GCA_001941865.1)**	30.6	87.97	87.49	0.12	0.81
***P*. *mendocina* PSB00032 (GCA_016008875.1)**	30.5	87.87	87.55	0.12	0.24
***Pseudomonas* sp. MY50 (GCA_009932725.1)** ^**a**^	30.4	88.09	87.79	0.12	0.53
***P*. *sihuiensis* KCTC 32246T (GCA_900106015.1)** ^**a**^	30.3	87.96	87.54	0.11	0.82
***P*. *chengduensis* DSM 26382 (GCA_900102635.1)** ^**a**^	30.3	87.93	87.95	0.12	1.03
***P*. *chengduensis* 402 (GCA_015712065.1)**	30.2	87.89	87.65	0.12	0.72
***P*. *mendocina* NCTC10897 (GCA_900636545.1)** ^**a**^	30.1	87.88	88.00	0.11	0.59
***Pseudomonas* sp. ZH-FAD (GCA_002803095.1)**	30.1	87.85	87.40	0.12	1.13
***P*. *alcaliphila* JCM 10630 (GCA_900101755.1)** ^**a**^	30.1	87.77	87.87	0.11	0.47
***P*. *toyotomiensis* JCM 15604 (GCA_900115695.1)** ^**a**^	30.0	87.84	87.79	0.12	0.74
***P*. *khazarica* TBZ2T (GCA_004521985.1)** ^**a**^	30.0	87.65	87.33	0.11	1.61
***Pseudomonas* sp. AOB-7 (GCA_003696305.1)**	30.0	87.66	86.01	0.12	3.27
***Pseudomonas* sp. 8Z (GCA_902506535.1)**	30.0	87.57	88.81	0.12	2.49
***P*. *mendocina* FFL34 (GCA_007049795.1)**	29.9	87.67	87.68	0.12	1.37
***P*. *composti* CCUG 59231 (GCA_900115475.1)** ^**a**^	29.8	87.66	87.97	0.12	0.94
***Pseudomonas* sp. 8O (GCA_902506495.1)**	29.7	87.57	87.50	0.12	0.88
***P*. *sediminis* PI11 (GCA_002741105.1)** ^**a**^	29.4	87.57	87.72	0.12	0.87
***Pseudomonas* sp. MSPm1 (GCA_014109765.1)**	29.3	87.31	87.10	0.12	0.94
***P*. *mendocina* NEB698 (GCA_003008615.1)**	29.2	87.34	87.00	0.12	0.92
***Azotobacter vinelandii* DSM 279 (GCA_900119555.1*)*** ^**b**^	22.4	84.43	72.67	0.19	2.13

^a^Type strain according to the NCBI database consulted on November 6, 2022.

^b^Selected genome as control outgroup.

**Fig 4:**
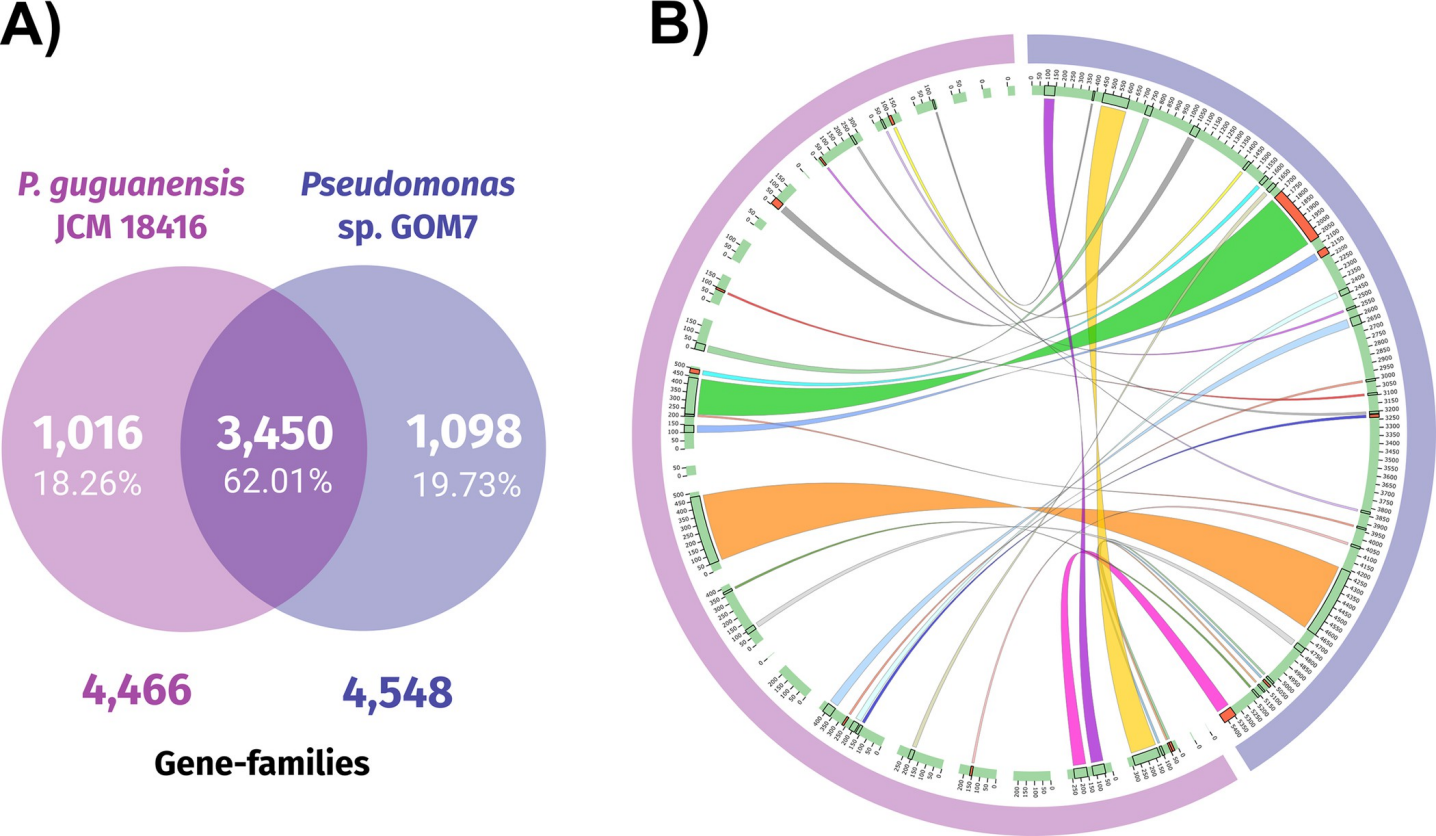
Analysis of gene families and comparison of multiple alignment blocks between the *Pseudomonas* sp. Comparison of the *Pseudomonas* sp. GOM7 (blue) and *P*. *guguanensis* JCM 18416 (purple) genomes. **A)** Venn diagram representing the distribution of shared and unique gene families between the two genomes. **B)** Genome synteny blocks obtained by Sibelia. From the outermost ring to the center, the featured ring contents are as follows: the two genomes are marked in blue and in purple, the contigs of each genome are marked in pale green, and the synteny blocks are represented by different colors. The red blocks in the contig ring indicate the reverse direction of the sequences.

The *P*. *guguanensis* JCM 18416 genome exhibited the highest dDDH (31.3%) and ANIm (88.30%) values (Table 2), indicating that this is the closest species to *Pseudomonas* sp. GOM7. The *Pseudomonas* sp. GOM7 and *P*. *guguanensis* JCM 18416 genomes share 3,450 gene families of the total 5,564 predicted by CMG BioTools (Fig 4A). CMG BioTools include in a gene family the genes whose proteins have 50% identity and 50% coverage [47]. The comparison of multiple alignment blocks (of minimum 10,000 bp length) between the two genomes showed 27 shared syntenic regions covering 29.21% of the *Pseudomonas* sp. GOM7 genome (Fig 4B). The synteny regions were distributed in 14 contigs of the *P*. *guguanensis* JCM 18416 genome, with a different order than in the *Pseudomonas* sp. GOM7 genome. The low genome coverage and the differences in the distribution of the synteny blocks indicate that the *Pseudomonas* sp. GOM7 genome has a dissimilar gene arrangement compared with the *P*. *guguanensis* JCM 18416 genome.

Together, these results show that *Pseudomonas* sp. GOM7 is a novel species within the *Pseudomonas* genus.

### Prediction of genes for possible antibacterial compounds in *Pseudomonas* sp. GOM7 genome

In agreement with the phenotypic assays showing no production of pyocyanin (S1 Fig), we found that the *Pseudomonas* sp. GOM7 genome lacks both large operons required for the biosynthesis of pyocyanin in *P*. *aeruginosa* (S3 Fig). However, *Pseudomonas* sp. GOM7 contains five genes showing identity with genes related to phenazine synthesis (*phzC*, *phzF*, *phzG*, *phzH*, and *phzS*); these genes are scattered throughout the genome and share the highest identities (84–97%) with putative genes of *Pseudomonas* sp. 8Z, *P*. *mendocina*, and *P*. *composti* strains (S3 Fig). Phenazines other than pyocyanin can also present antimicrobial activity [58]. Thus, it is tempting to speculate that the *phz* genes of *Pseudomonas* sp. GOM7 may be involved in the production of phenazines different from pyocyanin, or other molecules with antibacterial activity.

**Fig 5:**
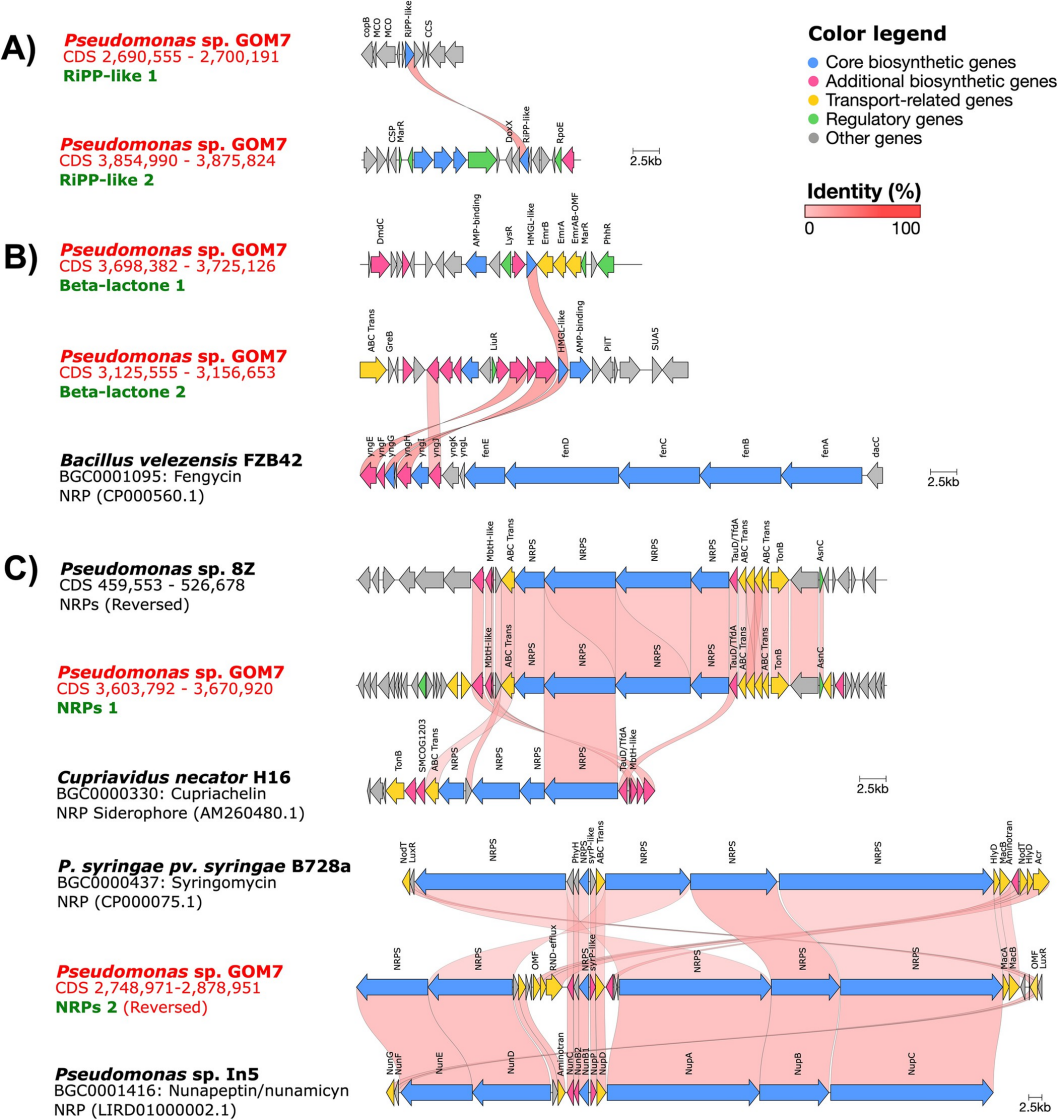
Predicted secondary metabolite biosynthetic gene clusters (BGCs) in the *Pseudomonas* sp. GOM7 genome and their comparison with BGCs from other bacteria. **A)** BGCs encoding for ribosomal-synthesized and posttranslationally modified peptides (RiPPs). **B)** BGCs encoding β-lactones. **C)** BGCs encoding ‘nonribosomal peptide synthetase modules’ (NRPs). Genes are represented as arrows, and the arrow direction indicates synteny. The probable function of genes and the identity between genes are indicated with colors according to the legends shown.

To identify other genes potentially encoding antimicrobial compounds, the *Pseudomonas* sp. GOM7 genome was analyzed with the antiSMASH program, which predicts biosynthetic gene clusters (BGCs) for secondary metabolites with probable antimicrobial activity. This analysis predicted six BGCs in the genome of *Pseudomonas* sp. GOM7 that could be involved in the production of antimicrobial compounds (Fig 5). Two of these BGCs are predicted to encode ribosomally synthesized and post-translationally modified peptides (RiPPs) (Fig 5A). Both BGCs contain a single RiPP-like gene, which presents 42% identity between them, and they do not share homology with other reported BGCs (Fig 5A). Two other BGCs of *Pseudomonas* sp. GOM7 are associated with the synthesis of potential β-lactones and share a biosynthetic HMGL-like gene that is 36% identical (Fig 5B). One of these later BGCs carries probable orthologs of the *yngEGHJ* genes of *Bacillus velezensis* FZB42, which are in an operon with the *fenABCDE* genes to synthesize the antifungal lipopeptide fengycin [59]; however, the *Pseudomonas* sp. GOM7 genome lacks the *fenABCDE* genes that are fundamental for fengycin production (Fig 5B). Two more BGCs of *Pseudomonas* sp. GOM7 were annotated as ‘nonribosomal peptide synthetase modules’ (NRPs) (Fig 5C). One of these NRPs contains probable orthologs for 17 of the 31 genes of a predicted NRP identified in the genome of the marine *Pseudomonas* sp. strain 8Z [60], with identities between 30–97% (Fig 5C). Additionally, this NRP of *Pseudomonas* sp. GOM7 also carries probable orthologs for 7 genes of *Cupriavidus necator* H16 involved in the production of the siderophore lipopeptide cupriachelin [61]; however, *Pseudomonas* sp. GOM7 lacks several other genes required for the synthesis of such compound (Fig 5C). The other NRP of *Pseudomonas* sp. GOM7 contains all genes required for the synthesis of the *P*. *syringae* syringomycin and *Pseudomonas fluorescens* nunapeptin/nunamicyn antimicrobial lipopeptides [62, 63], with identities between 31–82%, with the highest values for the nunapeptin/nunamicyn genes (Fig 5C).

These analyses revealed possible genes that may be involved in the antibacterial activity shown by *Pseudomonas* sp. GOM7.

The figure shows BGCs predicted with antiSMASH using the relaxed detection mode, when the strict detection mode was used the first BGC was not obtained.

### *Pseudomonas* sp. GOM7 does not show virulence properties in tested assays

**Fig 6:**
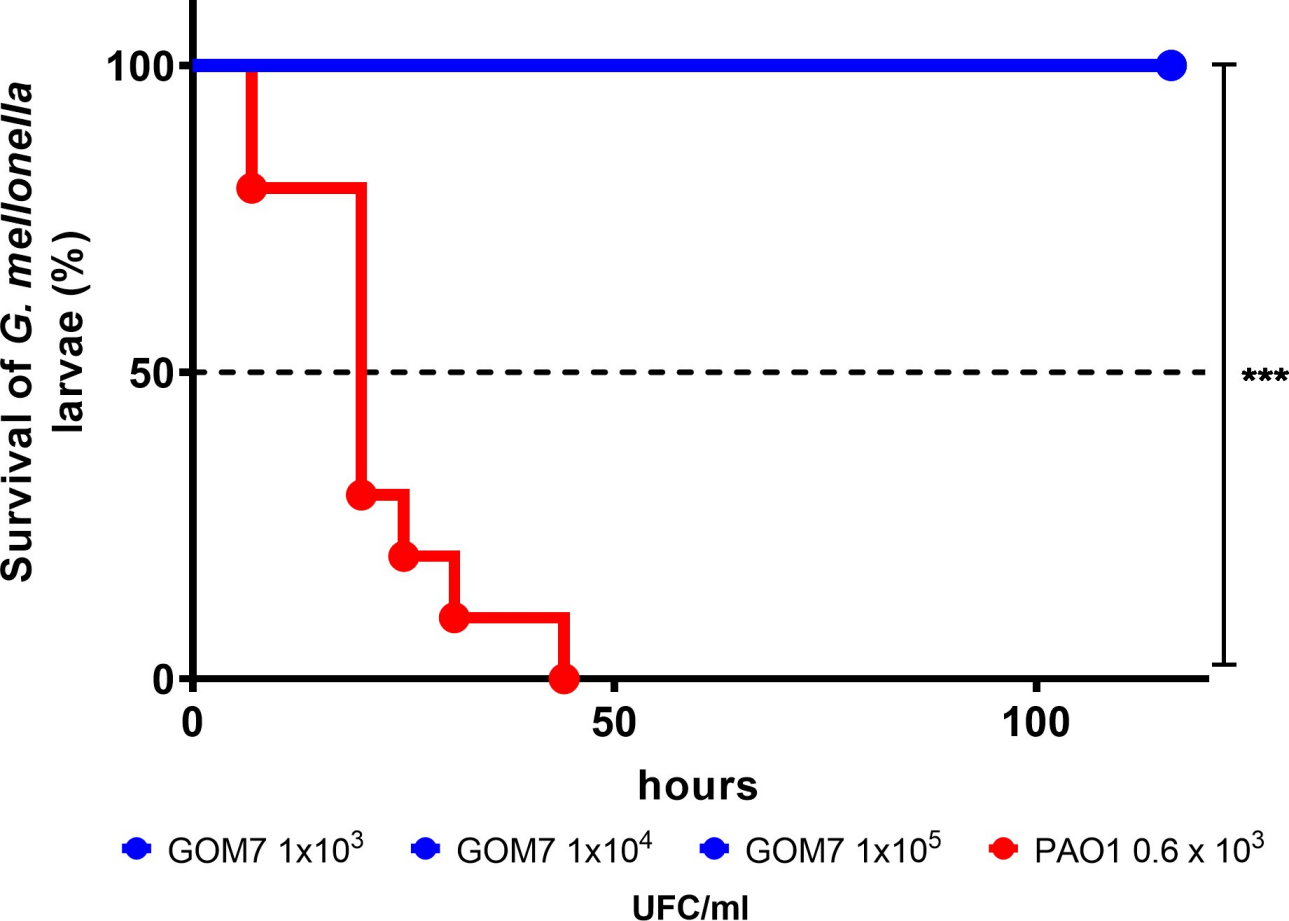
Survival of *G*. *mellonella* larvae. Mortality of *G*. *mellonella* larvae induced by bacterial infection. Kaplan-Meier survival curves were obtained by infecting larvae with the indicated CFUs of *Pseudomonas* sp. GOM7 (blue), *P*. *aeruginosa* PAO1 (red) and monitored daily for 5 days. Larvae not injected or injected only with 1X PBS did not kill any larvae. *P* value was calculated using the log-rank test. ***, *P* <0.001.

As an important trait to know for the study of a new bacterial species, we analyzed whether *Pseudomonas* sp. GOM7 shows different properties associated with the virulence of *P*. *aeruginosa*, the most relevant clinical species of *Pseudomonas*. First, the virulence of *Pseudomonas* sp. GOM7 and *P*. *aeruginosa* were compared in the *G*. *mellonella* infection model, which is widely used to test this phenotype [64]. As expected, *P*. *aeruginosa* killed 100% of the larvae at 40 h post-infection with an infective dose of 0.6 x 10^3^ CFUs. In contrast, *Pseudomonas* sp. GOM7 and the nonvirulent *E*. *coli* DH5α (used as a negative control) did not kill any larvae during the time tested, even with higher infective doses (1 x 10^3^, 1 x 10^4^, and 1 x10^5^ CFUs) (Fig 6). These results indicate that *Pseudomonas* sp. GOM7 is at least 167 times less virulent than *P*. *aeruginosa* in the *G*. *mellonella* model. Additionally, we compared *Pseudomonas* sp. GOM7 and *P*. *aeruginosa* for biofilm formation and the secretion of proteases, two phenotypes related to the virulence of *P*. *aeruginosa* [65]. As a negative control, the *E*. *coli* DH5α strain was also assessed. Our assays showed evident biofilm formation and secretion of proteases for *P*. *aeruginosa*; in contrast, *Pseudomonas* sp. GOM7 and *E*. *coli* DH5α strains presented a very low level of biofilm formation, ~5 and ~14 times lower than that of *P*. *aeruginosa*, respectively, and were negative for the secretion of protease test (S1 Fig). As described above, *Pseudomonas* sp. GOM7 does not produce pyocyanin (S1 Fig), an additional trait associated with *P*. *aeruginosa* virulence [66]. In agreement with these results, prediction of virulence factors in the *Pseudomonas* sp. GOM7 genome revealed the presence mostly of genes that can be associated with virulence but that are also present in nonpathogenic bacteria, such as genes for the synthesis and functioning of flagella and a Type IV pilus, as well as to produce alginate, lipopolysaccharide, and exopolysaccharide Ps1 (S5 Table). Overall, these results support that *Pseudomonas* sp. GOM7 is a nonpathogenic bacterium.

## Discussion

*Pseudomonas* is a ubiquitous genus that produces numerous molecules with different biological activities, such as antimicrobial compounds, siderophores, and lytic enzymes [67]. *Pseudomonas* species show high genetic and metabolic diversity, and most genes are species-specific or shared only among a subset of species, which contributes to the ability of these bacteria to colonize different environmental niches or hosts [17, 68].

In this study, we identified a new *Pseudomonas* species that we denominated *Pseudomonas* sp. GOM7, which was isolated from the GoM, did not show virulence properties in the assays tested and exhibited antibacterial activity. Genomic analysis for taxonomic identification indicates that the closest bacterial species to *Pseudomonas* sp. GOM7 is *P*. *guguanensis* JCM 18416 (Table 2), which was isolated from a hot spring water sample in Taiwan [69]. Four more of the 25 closest bacterial relatives to *Pseudomonas* sp. GOM7 were isolated from marine sources: *P*. *alcaliphila* JCM 10630 from seawater [70] and *P*. *chengduensis* 402, *Pseudomonas* sp. 8Z, and *Pseudomonas* sp. 8O from marine algae cultures [60]. Thus, the genome of *Pseudomonas* sp. GOM7 seems to contain traits of *Pseudomonas* species living in aquatic environments.

The colony and the culture supernatant of *Pseudomonas* sp. GOM7 inhibited the growth of *S*. *aureus*, including MDR strains and the priority pathogen MRSA (Table 1). Previous studies have reported other marine *Pseudomonas* species with antibacterial activity against *S*. *aureus*. For instance, *Pseudomonas* sp. UJ-6 and *Pseudomonas* sp. AMSN marine strains produce the compounds 1-acetyl-beta-carboline and 2,4-diacetylphloroglucinol (DAPG), respectively, which present antibacterial activity against MRSA [71, 72]. Furthermore, a *P*. *fluorescens* marine strain was found to produce the compounds andrimid and moiramides A-C, which also exhibit antibacterial activity against MRSA [73]. Additionally, the *P*. *alcaliphila* JCM 10630 marine strain produces toxoflavin, which has antibiotic properties against *Legionella pneumophila* and its host, the amoeba *Vermamoeba vermiformis* [74]. However, the *Pseudomonas* sp. GOM7 genome does not carry the genetic determinants associated with the production of toxoflavin nor any of the compounds described above. On the other hand, the *Pseudomonas* sp. GOM7 genome neither present the *flc* gene cluster (producing fluopsin C) that mediates antibacterial activity of *P*. *aeruginosa* against *S*. *aureus* [75].

The anti-*S*. *aureus* compound(s) produced by *Pseudomonas* sp. GOM7 was extracted from the culture supernatant of this bacterium with ethyl acetate (Fig 2). Many works have reported the suitability of ethyl acetate for the extraction of antibacterial compounds from bacteria, including marine strains [76, 77]. Compounds like peptides, fatty acids, esters, sterols, alkenes, and terpenes are soluble in ethyl acetate, [78–80]. Furthermore, metabolites like quinones, glycosides, alkaloids, and fatty acids with anti-MRSA activity have been identified in bacteria from ethyl acetate extractions [81, 82]. Computational analysis revealed six BGCs for secondary metabolites in the genome of *Pseudomonas* sp. GOM7; two seem to be specific for *Pseudomonas* sp. GOM7 (not orthologs were identified according to antiSMASH) and four present similarities with previously reported and characterized BGCs (Fig 5). The two specific BGCs of *Pseudomonas* sp. GOM7 encode RiPPs (Fig 5A), which are peptides abundant in the human microbiome and are recognized to have antibiotic activity [83]. Recently, a RiPP BGC was found and determined to be involved, along with ten other gene clusters, in the antibacterial activity of *Pseudomonas* sp. RGM2144 against the fish pathogen *Flavobacterium psychrophilum* [84]. Two more BGCs of *Pseudomonas* sp. GOM7 encode for β-lactones (Fig 5B), compounds that are related to a wide range of biological activities, including antibacterial, antifungal, and antiviral activities [85]. *Pseudomonas* sp. and *P*. *fluorescens* ATCC 39502 produce the β-lactones EM5395 and obafluorin (BGC0001437), respectively, which have activity against Gram-negative and Gram-positive bacteria [86, 87]. The two others predicted BGCs of *Pseudomonas* sp. GOM7 contain NRPs (Fig 5C). NRPs encode bioactive peptides and are found in bacteria, cyanobacteria, and fungi; NRPs producing antimicrobial peptides have been reported in marine bacteria [88]. One NRP of *Pseudomonas* sp. GOM7 is similar to a putative NRP from *Pseudomonas* sp. 8Z (Fig 5C) [60]. The other NRP of *Pseudomonas* sp. GOM7 showed the highest similarity with an NRP of the soil bacterium *P*. *fluorescens* In5, which synthesizes nunapeptin and nunamicyn, two peptides with antibacterial activity against the phytopathogen *Rhizoctonia solani* [63]. The same NRP of *Pseudomonas* sp. GOM7 also presented similarity to a NRP of the *P*. *syringae pv*. *syringae* B728a strain that synthesizes syringomycin, a compound with antibacterial activity against *Rhodococcus fascians*, *Micrococcus luteus*, *Rhodotorula pilimanae*, *Geotrichum candidum*, and the plant pathogen *Botrytis cinerea* [89]. Important to note, most predicted BGCs of *Pseudomonas* sp. GOM7 code for peptides, which are molecules that can be extracted with the solvent ethyl acetate [79]. Thus, it is tempting to speculate that the antibacterial activity of *Pseudomonas* sp. GOM7 is due to one or more peptides. Identification of the antibacterial compound(s) produced by *Pseudomonas* sp. GOM7 is a matter of our current investigation.

Our study provides further evidence showing the potential of marine microorganisms as a source of new antimicrobials.

## Supporting information

S1 Table Nonmarine bacterial strains used in this study. (PDF)

S2 Table Antibiotic susceptibility of *S*. *aureus* isolates. (PDF)

S3 Table Identification of marine isolates showing antibacterial activity by 16S rRNA sequencing. (PDF)

S4 Table Pyocyanin production by *P*. *aeruginosa* marine isolates showing antibacterial activity. (PDF)

S5 Table Virulence factors predicted for *Pseudomonas* sp. GOM7 by RASTtk annotation in the BV-BRC web server. (XLSX)

S1 Fig Comparison of virulence properties among *Pseudomonas* sp. GOM7, *P*. *aeruginosa* (pathogenic), and *E*. *coli* (nonpathogenic). **A)** Visualization of the characteristic blue-green coloration generated by pyocyanin on bacteria grown on cetrimide agar. **B)** Pyocyanin quantification in the supernatant of *Pseudomonas* sp. GOM7 and *P*. *aeruginosa* PAO1 cultures after sequential extraction with chloroform and quantification by spectrophotometry. **C)** Secretion of proteases. Production of extracellular proteases was evaluated in skim-milk agar. **D).** Biofilm formation. The crystal violet staining method was used to quantify biofilm formation. Quantification of pyocyanin production and biofilm formation was determined in bacteria grown in LB at 37°C. Bars represent averages ± S.D. *P* value was calculated using One-way ANOVA combined with Dunnett’s multiple comparison test. ***, *P* <0.001. *P*. *aeruginosa* PAO1 and *E*. *coli* DH5α strains were used as positive and negative controls, respectively. All assays were performed in triplicate.
(PDF)

S2 Fig *Pseudomonas* species closest to *Pseudomonas* sp. GOM7. **A)** Position of the *Pseudomonas* sp. GOM7 genome (red arrow) in the reference tree inferred by GTDB-Tk with FastTree v2.1.10 under the WAG model from the concatenated alignment of 120 ubiquitous bacterial gen. **B)** Clade of the reference tree where the *Pseudomonas* sp. GOM7 genome (in red) was placed together with genomes from other *Pseudomonas* species. The genome with the accession number in blue has been deleted from the NCBI.
(PDF)

S3 Fig Genes required for pyocyanin production in *P*. *aeruginosa* and some probable orthologous genes present in *Pseudomonas* sp. GOM7. **A)** The two clusters of genes that synthesize pyocyanin in *P*. *aeruginosa*. The enzymes encoded in the *phzA-phzG* genes transform chorismic acid into the phenazine-1-carboxylic acid (PCA), which is converted into different phenazines by the enzymes encoded in the *phzH*, *phzS*, and *phzM* genes; one phenazine is then transformed into pyocyanin by the enzyme encoded in the *phzS* gene. **B)** Probable *phzH*, *phzC*, *phzF*, *phzG*, and *phzS* orthologous genes present in the *Pseudomonas* sp. GOM7 genome. This figure was created with BioRender.com.
(PDF)
