# Supplementary material for: Genomic and phenotypic characterization of Pseudomonas sp. GOM7, a novel marine bacterial species with antimicrobial activity against multidrug-resistant Staphylococcus aureus
Source: PLoS One. 2023 Jul 13;18(7):e0288504. doi: 10.1371/journal.pone.0288504 (PMC10343084; doi:10.1371/journal.pone.0288504)
Supplement: S1 Table — (PDF) [file pone.0288504.s001.pdf]

S1 Table. Nonmarine bacterial strains used in this study

**Bacteria**	**Relevant features**
***Escherichia coli* ATCC 25922**	Reference strain
***Escherichia coli* DH10β**	Laboratory strain
***Escherichia coli* DH5α**	Laboratory strain
***Salmonella enterica* serotype Typhimurium SL1344**	Reference strain
***Klebsiella quasipneumoniae* ATCC 700603**	Reference strain
***Acinetobacter baumannii* ATCC 17978**	Reference strain
***Bacillus subtilis* 168**	Reference strain
***Pseudomonas aeruginosa* PAO1 (ATCC 15692)**	Reference strain.
***Enterococcus faecium* ATCC 19434**	Reference strain
***Staphylococcus aureus* ATCC 29213**	Reference strain
***S. aureus* ATCC 43300**	Reference strain, MRSA.
***S. aureus* 8N2**	Isolate from nasal exudate. MRSA.
***S. aureus* 4N34**	Isolate from nasal exudate,. MRSA.
***S. aureus* 14F4A**	Isolate from pharyngeal exudate, MRSA.
***S. aureus* 25F4**	Isolate from pharyngeal exudate, MRSA.
***S. aureus* 15N4**	Isolate from nasal exudate, MRSA.
***S. aureus* 1N3**	Isolate from nasal exudate, MRSA.
***S. aureus* 13F3**	Isolate from pharyngeal exudate, MRSA.
***S. aureus* 21F3**	Isolate from pharyngeal exudate, MRSA.
***S. aureus* 6N3**	Isolate from nasal exudate, MDR.
***S. aureus* 18F1**	Isolate from pharyngeal exudate, MDR.
***S. aureus* 24N2**	Isolate from nasal exudates, MDR.
***S. aureus* 25F2**	Isolate from pharyngeal exudate, MDR.
***S. aureus* 17F3**	Isolate from pharyngeal exudate, MDR.
***S. aureus* 17N3**	Isolate from nasal exudate, MDR.
