# Supplementary material for: Genomic and phenotypic characterization of Pseudomonas sp. GOM7, a novel marine bacterial species with antimicrobial activity against multidrug-resistant Staphylococcus aureus
Source: PLoS One. 2023 Jul 13;18(7):e0288504. doi: 10.1371/journal.pone.0288504 (PMC10343084; doi:10.1371/journal.pone.0288504)
Supplement: S2 Table — (PDF) [file pone.0288504.s002.pdf]

S2 Table. Antibiotic susceptibility of *S. aureus* isolates

***S. aureus* strains**	**CIP**	**FOS**	**TRS**	**P**	**VAN**	**TET**	**ERY**	**CLI**	**GEN**	**CEF**	**MIC for OXA**	**ARC**
**8N2**	S	S	S	R	S	R	R	R	R	S	8	MRSA
**4N3A**	S	S	S	R	S	S	S	R	S	S	8	MRSA
**14F4A**	S	S	S	R	S	S	R	S	S	S	4	MRSA
**25F4**	S	S	S	R	S	S	S	S	S	S	4	MRSA
**15N4**	S	S	S	R	S	S	R	R	S	S	32	MRSA
**1N3**	S	S	S	R	S	S	S	S	S	S	4	MRSA
**13F3**	S	S	S	R	S	S	S	S	S	S	4	MRSA
**21F3**	S	S	S	R	S	S	S	S	S	S	4	MRSA
**6N3**	S	S	S	R	S	R	R	S	S	S	2	MDR
**18F1**	S	S	S	R	S	S	R	R	S	S	2	MDR
**24N2**	S	S	S	R	S	R	R	R	R	S	2	MDR
**25F2**	S	S	S	R	S	R	R	R	S	S	<2	MDR
**17F3**	S	S	S	R	S	S	R	R	S	S	<2	MDR
**17N3**	S	S	S	R	S	S	R	R	S	S	<2	MDR

CIP= ciprofloxacin; FOS= fosfomycin; TRS= trimethoprim/sulfamethoxazole; P= penicillin; VAN= vancomycin; TET= tetracycline; ERY= erythromycin; CLI= clindamycin; GEN= gentamicin; CEF= cefalotin; ARC= antibiotic resistance classification; MRSA= Methicillin-resistant *Staphylococcus aureus*. MDR= Multidrug resistant. MIC (μg ml^{-1}) for oxacilin (OXA).
