# Supplementary material for: Genomic and phenotypic characterization of Pseudomonas sp. GOM7, a novel marine bacterial species with antimicrobial activity against multidrug-resistant Staphylococcus aureus
Source: PLoS One. 2023 Jul 13;18(7):e0288504. doi: 10.1371/journal.pone.0288504 (PMC10343084; doi:10.1371/journal.pone.0288504)
Supplement: S3 Table — (PDF) [file pone.0288504.s003.pdf]

S3 Table. Identification of marine isolates showing antibacterial activity by sequencing of the 16S rRNA gene.

Isolate	Organism best match	Query cover (%)	E value	Identity (%)	Accession Number
**GOM7**	*P. sihuiensis* strain WM-2	100	0	98.94	NR_148251.1
**GOM1**[a]	*P. aeruginosa* strain DSM 50071	ND	0	99.93	NR_117678.1
**LP17**	*P. aeruginosa* strain DSM 50071	100	0	100	NR_117678.1
**LP21**	*P. aeruginosa* strain DSM 50071	100	0	100	NR_117678.1
**LP34**	*P. aeruginosa* strain DSM 50071	99	0	99.71	NR_117678.1
**LP35**	*P. aeruginosa* strain DSM 50071	99	0	99.76	NR_117678.1
**LP36**	*P. aeruginosa* strain DSM 50071	96	0	99.14	NR_117678.1
**LP89**	*P. aeruginosa* strain DSM 50071	99	0	99.66	NR_117678.1

[a]*P. aeruginosa* marine isolate described in Muriel-Millán et al., 2019 [20].
