# Supplementary material for: Genomic and phenotypic characterization of Pseudomonas sp. GOM7, a novel marine bacterial species with antimicrobial activity against multidrug-resistant Staphylococcus aureus
Source: PLoS One. 2023 Jul 13;18(7):e0288504. doi: 10.1371/journal.pone.0288504 (PMC10343084; doi:10.1371/journal.pone.0288504)
Supplement: S4 Table — (PDF) [file pone.0288504.s004.pdf]

**S4 Table.** Pyocyanin production by *P. aeruginosa* marine isolates showing antibacterial activity.

| **Isolate** | **Pyocyanin production[b] (μg ml$^{-1}$)** |
|---|---|
| **GOM1[a]** | 4.82 ± 0.392 |
| **LP17** | 3.02 ± 0.141 |
| **LP21** | 2.24 ± 0.119 |
| **LP34** | 5.06 ± 0.136 |
| **LP35** | 6.18 ± 0.096 |
| **LP36** | 5.45 ± 0.472 |
| **LP89** | 4.44 ± 0.328 |

[a]*P. aeruginosa* marine isolate described in Muriel-Millán et al., 2019 [20].

[b]The data are presented as the mean ± S.D.
