# Supplementary material for: Genomic and phenotypic characterization of Pseudomonas sp. GOM7, a novel marine bacterial species with antimicrobial activity against multidrug-resistant Staphylococcus aureus
Source: PLoS One. 2023 Jul 13;18(7):e0288504. doi: 10.1371/journal.pone.0288504 (PMC10343084; doi:10.1371/journal.pone.0288504)
Supplement: S1 Fig — A) Visualization of the characteristic blue-green coloration generated by pyocyanin on bacteria grown on cetrimide agar. B) Pyocyanin quantification in the supernatant of Pseudomonas sp. GOM7 and P. aeruginosa PAO1 cultures after sequential extraction with chloroform and quantification by spectrophotometry. C) Secretion of proteases. Production of extracellular proteases was evaluated in skim-milk agar. D). Biofilm formation. The crystal violet staining method was used to quantify biofilm formation. Quantification of pyocyanin production and biofilm formation was determined in bacteria grown in LB at 37°C. Bars represent averages ± S.D. P value was calculated using One-way ANOVA combined with Dunnett’s multiple comparison test. ***, P <0.001. P. aeruginosa PAO1 and E. coli DH5α strains were used as positive and negative controls, respectively. All assays were performed in triplicate. (PDF) [file pone.0288504.s006.pdf]

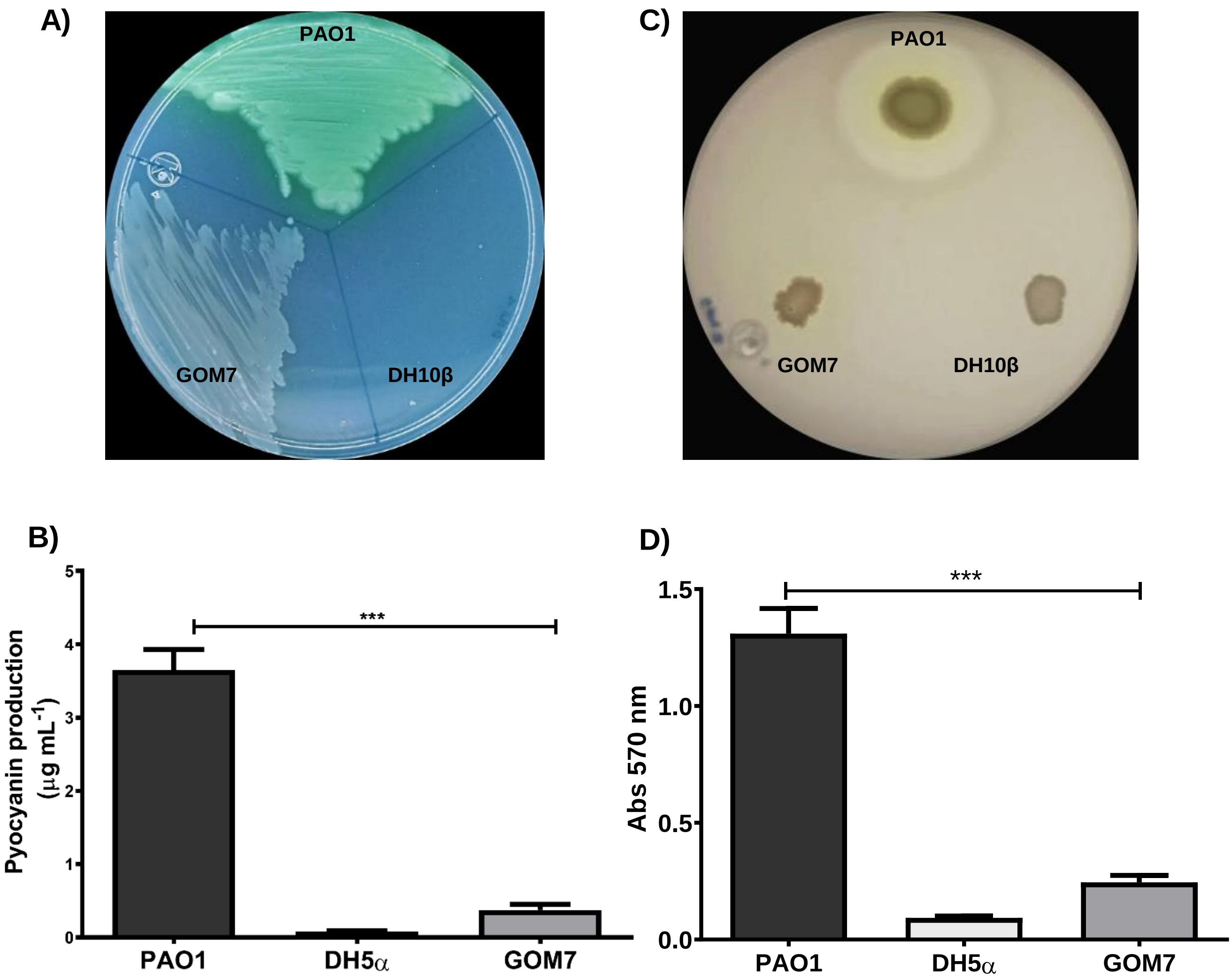

S1 Fig. Comparison of virulence properties among *Pseudomonas* sp. GOM7, *P. aeruginosa* (pathogenic), and *E. coli* (nonpathogenic). A) Visualization of the characteristic blue-green coloration generated by pyocyanin on bacteria grown on cetrimide agar. **B)** Pyocyanin quantification in the supernatant of *Pseudomonas* sp. GOM7 and *P. aeruginosa* PAO1 cultures after sequential extraction with chloroform and quantification by spectrophotometry. **C)** Secretion of proteases. Production of extracellular proteases was evaluated in skim-milk agar. **D)**. Biofilm formation. The crystal violet staining method was used to quantify biofilm formation. Quantification of pyocyanin production and biofilm formation was determined in bacteria grown in LB at 37 °C. Bars represent averages ± S.D. *P* value was calculated using One-way ANOVA combined with Dunnett's multiple comparision test. ***, *P* <0.001. *P. aeruginosa* PAO1 and *E. coli* DH5α strains were used as positive and negative controls, respectively. All assays were performed in triplicate.
