# Supplementary material for: Genomic and phenotypic characterization of Pseudomonas sp. GOM7, a novel marine bacterial species with antimicrobial activity against multidrug-resistant Staphylococcus aureus
Source: PLoS One. 2023 Jul 13;18(7):e0288504. doi: 10.1371/journal.pone.0288504 (PMC10343084; doi:10.1371/journal.pone.0288504)
Supplement: S2 Fig — A) Position of the Pseudomonas sp. GOM7 genome (red arrow) in the reference tree inferred by GTDB-Tk with FastTree v2.1.10 under the WAG model from the concatenated alignment of 120 ubiquitous bacterial gen. B) Clade of the reference tree where the Pseudomonas sp. GOM7 genome (in red) was placed together with genomes from other Pseudomonas species. The genome with the accession number in blue has been deleted from the NCBI. (PDF) [file pone.0288504.s007.pdf]

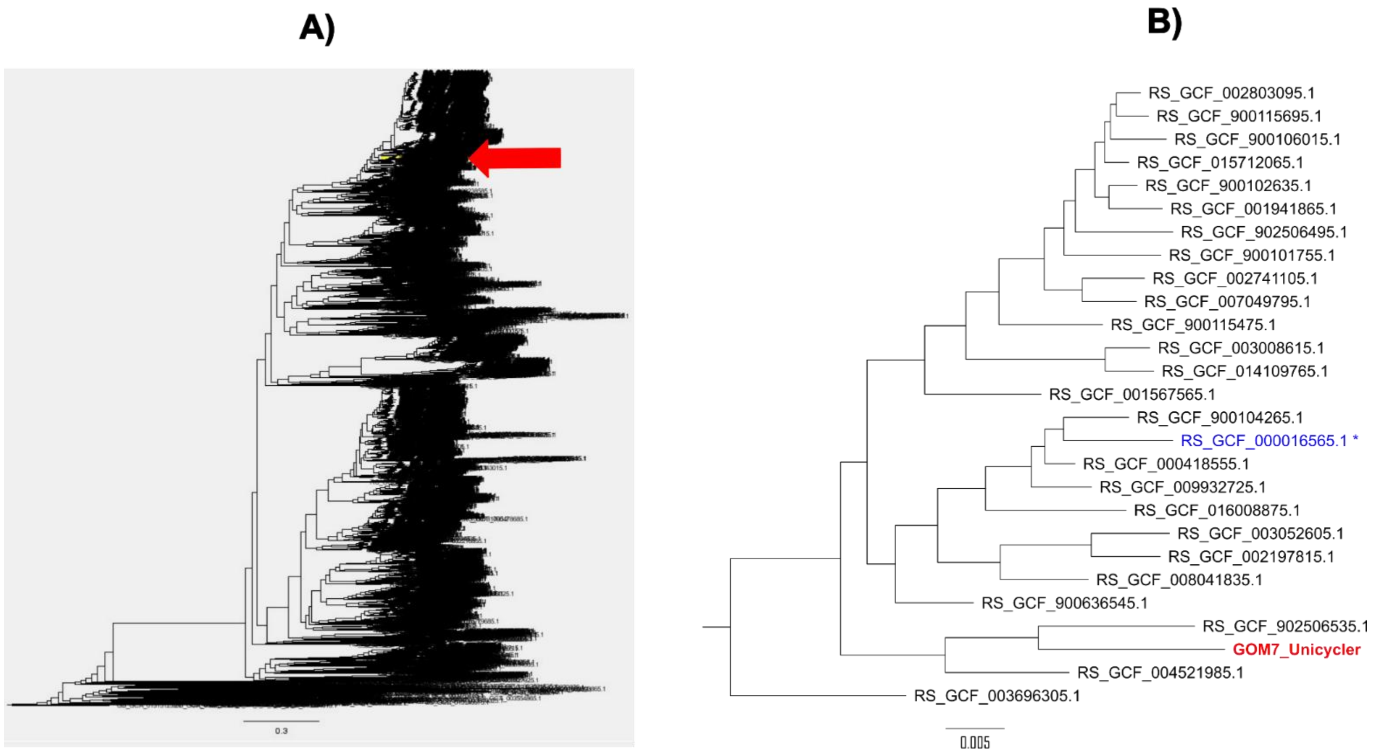

S2 Fig. *Pseudomonas* species closest to *Pseudomonas* sp. GOM7. **A)** Position of the *Pseudomonas* sp. GOM7 genome (red arrow) in the reference tree inferred by GTDBTk with FastTree v2.1.10 under the WAG model from the concatenated alignment of 120 ubiquitous bacterial genes. **B)** Clade of the reference tree where the *Pseudomonas* sp. GOM7 genome (in red) was placed together with genomes from other *Pseudomonas* species. The genome with the accession number in blue has been deleted from the NCBI.
