# Supplementary material for: Genomic and phenotypic characterization of Pseudomonas sp. GOM7, a novel marine bacterial species with antimicrobial activity against multidrug-resistant Staphylococcus aureus
Source: PLoS One. 2023 Jul 13;18(7):e0288504. doi: 10.1371/journal.pone.0288504 (PMC10343084; doi:10.1371/journal.pone.0288504)
Supplement: S3 Fig — A) The two clusters of genes that synthesize pyocyanin in P. aeruginosa. The enzymes encoded in the phzA-phzG genes transform chorismic acid into the phenazine-1-carboxylic acid (PCA), which is converted into different phenazines by the enzymes encoded in the phzH, phzS, and phzM genes; one phenazine is then transformed into pyocyanin by the enzyme encoded in the phzS gene. B) Probable phzH, phzC, phzF, phzG, and phzS orthologous genes present in the Pseudomonas sp. GOM7 genome. This figure was created with BioRender.com. (PDF) [file pone.0288504.s008.pdf]

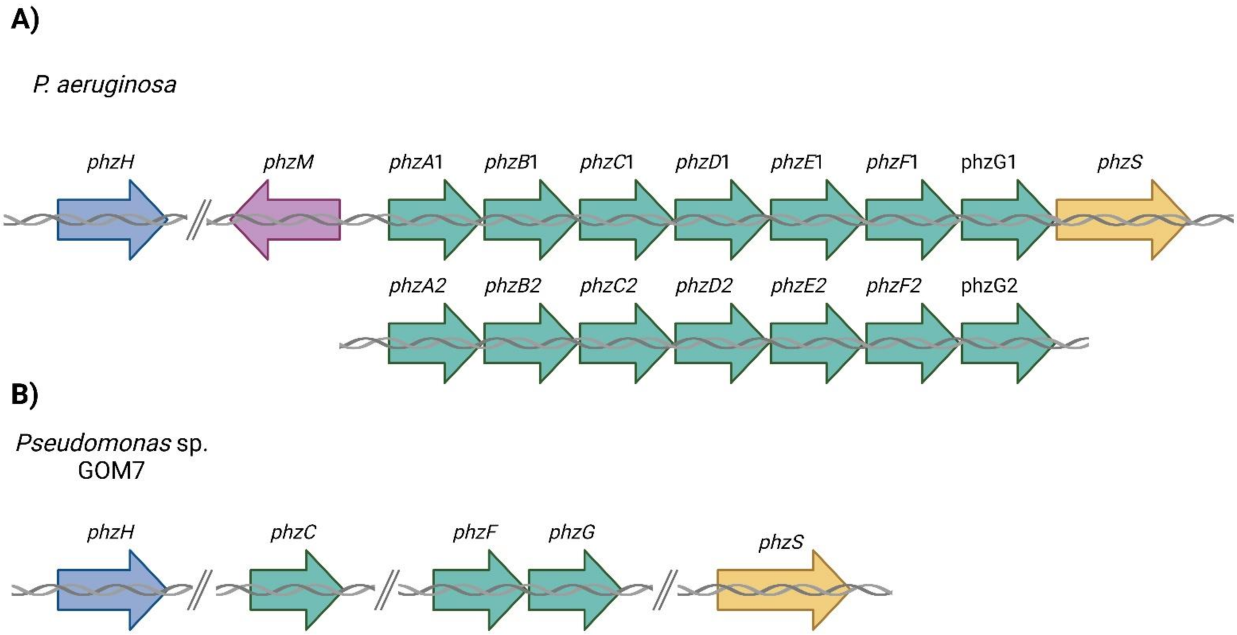


**S3 Fig. Genes required for pyocyanin production in *P. aeruginosa* and some probable orthologous genes present in *Pseudomonas* sp. GOM7**. **A)** The two clusters of genes that synthesize pyocyanin in *P. aeruginosa*. The enzymes encoded in the *phzA-phzG* genes transform chorismic acid into the phenazine-1-carboxylic acid (PCA), which is converted into different phenazines by the enzymes encoded in the *phzH*, *phzS*, and *phzM* genes; one phenazine is then transformed into pyocyanin by the enzyme encoded in the *phzS* gene. **B)** Probable *phzH, phzC, phzF, phzG,* and *phzS* orthologous genes present in the *Pseudomonas* sp. GOM7 genome. This figure was created with BioRender.com.
